# CD58 expression does not impact response to inotuzumab ozogamicin in patients with B‐cell acute lymphoblastic leukemia

**DOI:** 10.1002/jha2.1076

**Published:** 2024-12-28

**Authors:** Rafael Madero‐Marroquin, Ryan W. Hunter, Caner Saygin, Hannah Johnston, Adam S. DuVall, Hamed Rahmani Youshanlouei, Clinton Osei, Syed Shah, Wendy Stock, Sandeep Gurbuxani, Anand A. Patel

**Affiliations:** ^1^ Department of Medicine, Section of Hematology/Oncology University of Chicago Chicago Illinois USA; ^2^ Department of Pathology University of Chicago Chicago Illinois USA

**Keywords:** acute leukemia, ALL, CD58, inotuzumab ozogamicin

## Abstract

**Background:**

CD58 loss has been described as a mechanism of resistance to blinatumomab and chimeric antigen receptor T‐cell therapy, functioning as a modulator of response to T‐cell activation.

**Methods:**

Using flow cytometry, we evaluated the impact of CD58 mean fluorescence intensity (MFI) on the probability of achieving measurable residual disease (MRD) negativity in patients with B‐cell acute lymphoblastic leukemia treated with inotuzumab ozogamicin (InO).

**Results:**

The odds ratio of achieving MRD negativity was 1.03 for every 1000 unit increase in CD58 MFI.

**Conclusion:**

Our results suggest that MRD negativity rates after InO are high, regardless of the intensity of CD58 expression.

## 1 INTRODUCTION

Therapeutic advancements over recent decades have improved clinical outcomes for B‐cell acute lymphoblastic leukemia (B‐ALL) [1]. This improvement has been driven in part by the development of new antibody‐based therapies, such as blinatumomab and inotuzumab ozogamicin (InO), with InO currently Food and Drug Administration‐approved for use in relapsed and refractory (R/R) B‐ALL [2, 3]. These antibody‐based therapies are now being studied in the frontline setting due to their proven efficacy in the R/R setting, as well as their favorable tolerance in the elderly and frail population when compared to intensive chemotherapy regimens [4]. As these treatments become more widely utilized, the identification of resistance mechanisms and predictive markers of response have become topics of interest [5, 6].

CD58 has been identified as a modulator of response to blinatumomab and chimeric antigen receptor T‐cell (CAR‐T) therapy, functioning as a CD2 ligand during T‐cell activation; loss of CD58 expression is a mechanism of resistance to these therapies [7, 8]. This interaction between CD58 and CD2 has also been described as a potentiator of cellular lysis mediated by natural killer cells (NK cells) [9]. While reported mechanisms of resistance to InO include the development of CD22 epitope loss or CD22 epitope alterations, the impact of CD58 expression has not been investigated [5]. Given that NK‐cell mediated lysis is a proposed mechanism of action of antibody‐drug conjugates, such as InO, we studied the role of CD58 expression as a predictor of morphologic response to InO [10].

## 2 METHODS

We conducted a retrospective chart review to identify patients in our institutional B‐ALL database who received InO between September 15, 2011, and March 10, 2023. Patients were excluded if they did not have adequate blood or bone marrow flow cytometry samples available before initiating InO or if they were in morphologic complete remission (CR) immediately before receiving InO. Multicolor flow cytometry was performed on patient samples, run on a Beckman Coulter flow cytometer. Data were subsequently analyzed with Kaluza software (Beckman Coulter). After gating for live singlets by forward and side scatter properties, the CD45 versus side scatter gating strategy was employed to categorize populations, including blasts (dim CD45 and low side scatter) and lymphocytes (bright CD45 and low side scatter). CD58 thresholds were set based on populations of CD58‐negative mature B‐lymphocytes. Consistent overexpression of CD58 was observed on leukemic blasts, in accordance with previous reports [11, 12]. Therefore, as the percentage of blasts expressing CD58 would not be a discriminatory metric, we instead utilized geometric mean fluorescence intensity (MFI) for subsequent comparisons. We also reported the MFI of CD22, which is the primary target of InO and was evaluated in all patients prior to treatment [13]. Morphologic response assessment and risk stratification were performed using the National Comprehensive Cancer Network (NCCN) guidelines [14]. We considered a morphologic response as the achievement of CR, CR with partial hematologic recovery (CRh), or with incomplete hematologic recovery (CRi). Measurable residual disease (MRD) was evaluated using flow cytometry with a sensitivity of 10^−4^. We evaluated median overall survival (OS) and leukemia‐free survival (LFS) in our cohort; we defined the latter as the time between the date of morphologic remission and the date of relapse or last follow‐up.

To evaluate the impact of CD58 MFI on treatment outcomes, we performed a simple logistic regression analysis evaluating the probability of having MRD negativity according to CD58 MFI. In this model, we considered MRD as a dependent dichotomous variable and CD58 MFI as an independent continuous variable. To account for outlier samples of CD58 MFI expression, we verified our results by dividing our cohort into CD58 MFI‐high and CD58 MFI‐low according to their CD58 MFI expression above or below the median, respectively. We then compared the rates of MRD negativity between CD58 MFI‐high and CD58 MFI‐low groups by using Fisher's exact test. Since InO is a CD22‐directed antibody‐drug conjugate, we repeated this analysis using CD22 MFI and compared response rates between CD22 MFI‐high and CD22 MFI‐low patients. To evaluate the impact of individual variables of interest on patient responses, we calculated the odds of achieving morphologic remission according to age (≥ 35 years old), molecular risk at diagnosis, and R/R disease status. The statistical analysis was performed using R version 4.3.1.

## 3 RESULTS

We identified 75 patients who received InO at our institution. Thirty‐five patients did not have an adequate flow cytometry sample at our institution prior to their first dose of InO. Forty patients had adequate flow cytometry samples immediately prior to treatment initiation with InO. Six of these patients were excluded because they received InO while in morphologic CR, whether as part of consolidation or due to MRD positivity. A total of 34 patients met the criteria for inclusion in our analysis; two of these patients were previously treated with InO. Patient and disease characteristics for this cohort can be found in Table 1. Twenty‐eight patients (82%) were treated with single‐agent InO, four patients (12%) with InO plus dasatinib, one patient (3%) with InO plus vincristine, and one patient (3%) with InO plus venetoclax. Only one patient had a dose reduction of InO using a day 1 dose of 0.5 mg/m^2^ due to concern for liver toxicity, but still achieved CR with MRD negativity.

**TABLE 1:** Patient characteristics.

	***N* = 34**
Median age at the time of InO infusion (range), years	58.5 (16–78)
**Gender, number (%)**	
Female	19 (55.9%)
Male	15 (44.1%)
**Race/Ethnicity, number (%)**	
Non‐Hispanic White	19 (55.9%)
Hispanic	9 (26.5%)
Non‐Hispanic Black	5 (14.7%)
Non‐Hispanic Asian	1 (2.9%)
**ECOG Performance Status at the time of InO infusion, number (%)** ^a^	
0	13 (38.2%)
1	15 (44.1%)
2	5 (14.7%)
**B‐ALL characteristics, number (%)**	
Patients with poor‐risk cytogenetics at diagnosis^b^	13 (38.2%)
Patients with therapy‐related leukemia	8 (23.5%)
**Flow cytometry characteristics, median (range)**	
CD58 %	99.93% (12.04%–100%)
CD58 MFI	11,719. 88 (1959.85–55,217.14)
CD22 %	88.53% (3.32–99.59%)
CD22 MFI	4,516.14 (174.22–21,825.07)
**Indication for InO, number (%)**	
First‐line	16 (47.1%)
Relapsed/refractory disease	18 (52.9%)
**InO response, number (%)**	
CR/CRi	30 (88.2%)
MRD‐negative	27 (79.4%)

Abbreviations: B‐ALL = B‐cell Acute Lymphoblastic Leukemia; CR = Complete Remission; CRi = Complete Remission with incomplete blood count recovery; ECOG = Eastern Cooperative Oncology Group; InO = Inotuzumab Ozogamicin; MFI = Mean Fluorescence Intensity; MRD = Measurable Residual Disease.

^a^ Performance status data not available for one patient.

^b^ Diagnostic cytogenetic data not available for two patients.

Our cohort had a median OS of 21.5 months (range, 1–58 months), with a median LFS of 13.5 months (range, 1–52 months). A total of 30 patients (88.2%) had a complete morphologic remission, of which 28 patients (82.4%) had a CR, two patients (5.9%) had a CRi, and 27 patients (79.4%) had no MRD by flow cytometry. The two patients who had a history of prior treatment with InO had a CR with MRD negativity. Of the four patients (11.8%) who did not have a response to InO, one patient had a diagnosis of chronic myeloid leukemia (CML) in lymphoid blast crisis.

**FIGURE 1:**
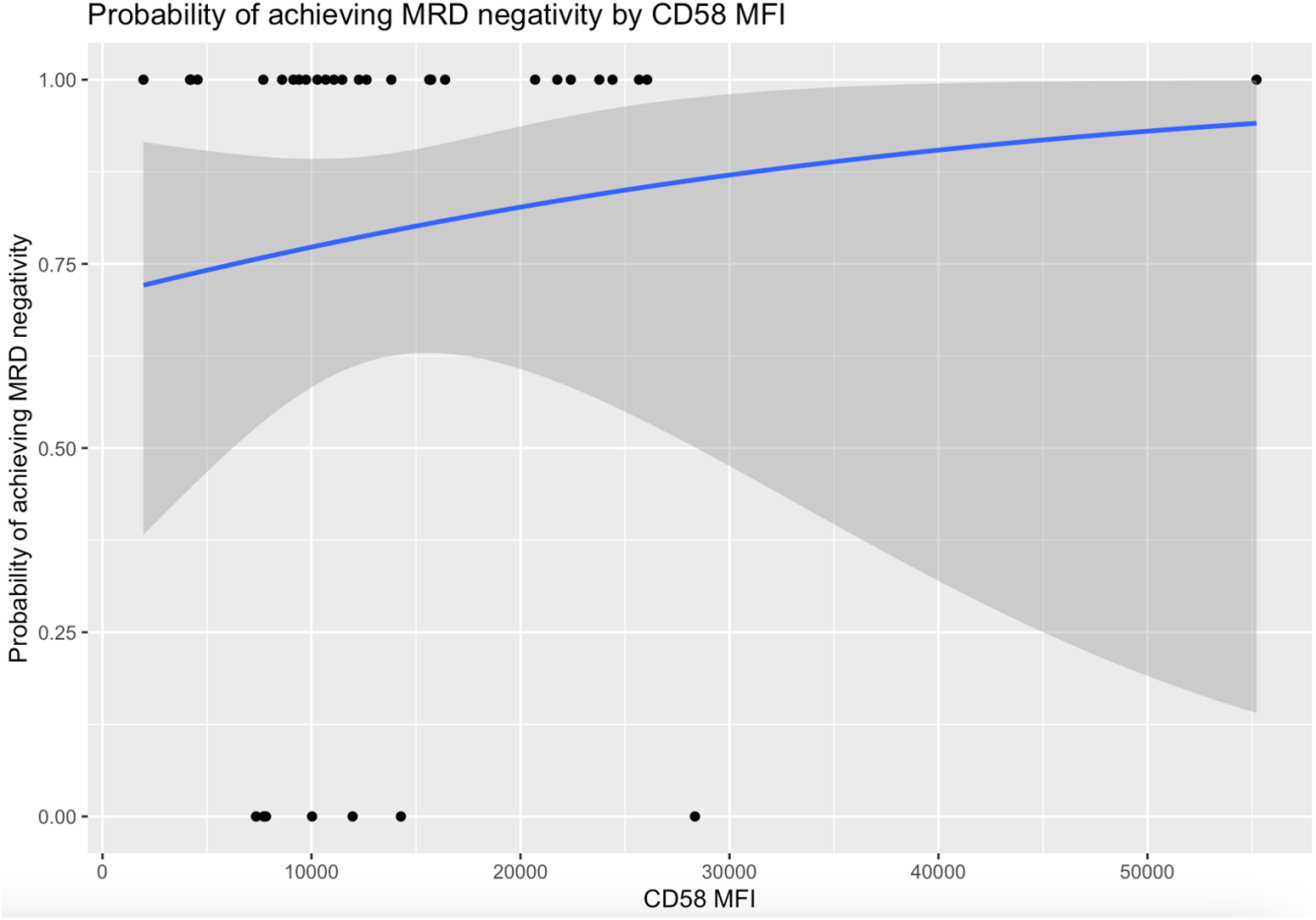
Logistic regression curve predicting the probability of achieving MRD negativity according to CD58 MFI. Sample points are marked with black dots, plotted across the y‐axis depending on the presence (0) or absence [1] of MRD after treatment with inotuzumab ozogamicin. The dark gray area represents 95% confidence intervals for the logistic regression curve. MRD: measurable residual disease; MFI: mean fluorescence intensity.

Our simple logistic regression model (Figure 1), using CD58 MFI as a predictor for MRD negativity, did not achieve statistical significance when using the effect likelihood ratio test (*p *= 0.495). The odds ratio (OR) of achieving MRD negativity was 1.03 for every 1000 unit increase in CD58 MFI. These findings were reiterated when comparing the outcomes between patients who had CD58 MFI‐high versus CD58 MFI‐low B‐ALL. Three patients out of 17 had MRD positivity in the CD58 MFI‐high group, compared to four patients out of 17 in the CD58 MFI‐low group. No statistically significant difference was observed between these groups (*p *= 1.000). A comparison between CD22 MFI‐high and CD22 MFI‐low patients showed no statistical difference between response rates in each group (*p *= 0.398). A total of two out of 17 patients had MRD in the CD22 MFI‐high group and five out of 17 patients had MRD in the CD22 MFI‐low group. In our univariate analysis, we did not identify a statistically significant difference in the odds of having persistent MRD after InO when accounting for poor molecular risk at diagnosis (OR = 3.79, 95% confidence interval [CI 0.58, 24.76]), age > 35 years (OR = 0.47, 95% CI [0.08, 2.62]), or R/R disease (OR = 7.5, 95% CI [0.79, 71.09]).

## 4 DISCUSSION

Patients in this cohort achieved high rates of response to InO, with a CR/CRi rate of 88.2% and an MRD negativity rate of 79.4% by flow cytometry. Using our simple logistic regression model, the probability of MRD negativity after InO was high irrespective of the intensity of CD58 expression as measured by MFI. These results were corroborated after comparing response rates in CD58 MFI‐high and CD58 MFI‐low patients, suggesting that CD58 expression in B‐ALL does not impact the probability of MRD negativity after treatment with InO.

These data suggest that InO remains an effective therapeutic option for B‐ALL regardless of the intensity of CD58 expression. It is important to consider these findings given the previously described role of CD58 loss as a possible mechanism of resistance to CAR‐T and blinatumomab therapy [7, 8]. Despite the use of InO as frontline therapy in current clinical trials, experience at our center suggests that InO remains an effective option in R/R B‐ALL patients with prior treatment with InO [4, 15]. Our study is limited by a small sample size, as well as a retrospective and single‐center design. It is possible that the study was underpowered to detect a statistically significant difference in MRD response according to CD58 MFI. Additional studies are needed to further evaluate the utility of CD58 as a predictive marker of response to InO and other B‐ALL treatment options, including blinatumomab and CAR‐T.

## AUTHOR CONTRIBUTIONS

Rafael Madero‐Marroquin collected the clinical data, analyzed the data, and drafted the initial manuscript; Ryan W. Hunter collected and analyzed flow cytometry data; Caner Saygin, Wendy Stock, Sandeep Gurbuxani, and Anand A. Patel conceptualized the project; Anand A. Patel and Sandeep Gurbuxani supervised the project; all authors reviewed and edited the finished manuscript.

## CONFLICT OF INTEREST STATEMENT

Adam S. DuVall: speaker for CE Concepts. Wendy Stock: advisor for Kura, Servier, Newave, and Asofarma.

Sandeep Gurbuxani: consulting or advisory role: AbbVie, Jazz Pharmaceuticals. Patents, Royalties, Other Intellectual Property: Royalties from UpToDate for contributions to various topics.

Anand A. Patel: honoraria from AbbVie, Bristol Myers Squibb, and Sobi; research funding (institutional) from Pfizer, Kronos Bio, and Sumitomo. The rest of the authors declare no conflict of interest.

## FUNDING INFORMATION

N/A

## ETHICS STATEMENT

All patients provided consent to have clinical information reported via our institutional leukemia registry, which was approved by our internal review board.

## PATIENT CONSENT STATEMENT

The authors have confirmed patient consent statement is not needed for this submission.

## CLINICAL TRIAL REGISTRATION

The authors have confirmed clinical trial registration is not needed for this submission

## Data Availability

Given concerns for medical privacy, data will not be made publicly available. For original data, please contact anand.patel@bsd.uchicago.edu.
